# InChI isotopologue and isotopomer specifications

**DOI:** 10.1186/s13321-024-00847-8

**Published:** 2024-05-14

**Authors:** Hunter N. B. Moseley, Philippe Rocca-Serra, Reza M. Salek, Masanori Arita, Emma L. Schymanski

**Affiliations:** 1 Department of Molecular & Cellular Biochemistry, University of Kentucky, Lexington, KY USA; 2 Department of Engineering Science, University of Oxford E-Research Centre, University of Oxford, Oxford, OX1 3QG UK; 3 BiotechVision, Cambridge, UK; 4 Bioinformation and DDBJ Center, National Institute of Genetics, Mishima, Shizuoka 411-8540 Japan; 5 Luxembourg Centre for Systems Biomedicine (LCSB), University of Luxembourg, 6 Avenue du Swing, 4367 Belvaux, Luxembourg

**Keywords:** International chemical identifier, InChI, Isotopologue, Isotopomer, Chemical annotation, Isotope-resolved

## Abstract

**Abstract:**

This work presents a proposed extension to the International Union of Pure and Applied Chemistry (IUPAC) International Chemical Identifier (InChI) standard that allows the representation of isotopically-resolved chemical entities at varying levels of ambiguity in isotope location. This extension includes an improved interpretation of the current isotopic layer within the InChI standard and a new isotopologue layer specification for representing chemical intensities with ambiguous isotope localization. Both improvements support the unique isotopically-resolved chemical identification of features detected and measured in analytical instrumentation, specifically nuclear magnetic resonance and mass spectrometry.

**Scientific contribution:**

This new extension to the InChI standard would enable improved annotation of analytical datasets characterizing chemical entities, supporting the FAIR (Findable, Accessible, Interoperable, and Reusable) guiding principles of data stewardship for chemical datasets, ultimately promoting Open Science in chemistry.

## Introduction

The International Union of Pure and Applied Chemistry (IUPAC) International Chemical Identifier (InChI) is an ASCII character string for uniquely identifying a chemical compound [1–3]. The InChI generation algorithm utilizes an augmented (highly modified) McKay’s canonical graph labeling algorithm [4, 5] for the purpose of uniquely numbering atoms in a chemical compound graph regardless of the tautomer representation used in the InChI generation. The InChI generation algorithm was originally developed and implemented in the C programming language by the InChI Subcommittee of the IUPAC Chemical Nomenclature and Structure Representation Division (Division VIII). Subsequently in 2009, the InChI Trust, an international organization, was established to oversee the development of the algorithm and its implementations, leaving the IUPAC Division VIII InChI Subcommittee to oversee the conceptual design and specification of the InChI standard itself [6]. The current InChI libraries and software are freely available under the IUPAC-InChI Trust open source license [7] and includes indirect multi-language support through a compiled library that provides an application programming interface (API) [3]. Over the years, the InChI generation algorithm and software have been augmented to recognize more tautomeric forms of chemical compounds [3] as well as provide additional chemical description in the form of additional layers within the InChI string. InChI is also incorporated into larger identifier strings that represent other chemical phenomena including reactions (RInChI) [8], mixtures (MInChI) [9, 10], nanomaterials (NInChI) [11], and variable R-groups (VInChI and MarkInChI) [3].

**Fig. 1:**
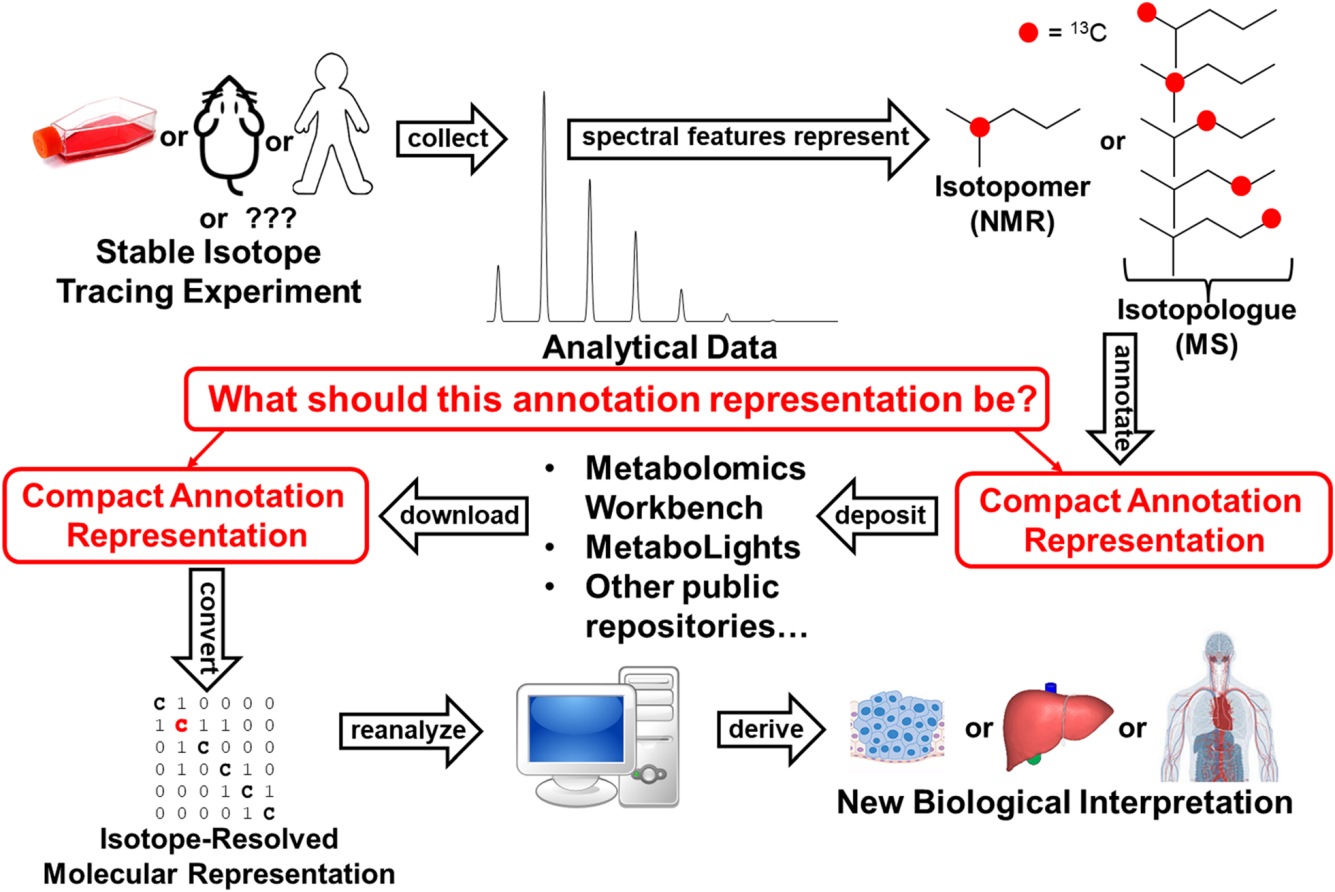
Overview of the isotopically-resolved spectral feature annotation challenge. First, samples are taken from biological entities in stable isotope tracing experiments. Next, analytical data representing nuclear magnetic resonance (NMR) derived isotopomer spectral features and mass spectrometry (MS) isotopologue spectral features is collected. Spectral features require isotopically-resolved annotation before deposition into a scientific repository for later retrieval and reanalysis for deriving new biological interpretation within different experimental contexts. How can we best represent isotopically-resolved annotations? What should this annotation representation be?

In its current form, the InChI standard cannot be used to accurately annotate spectral features for isotopically-resolved chemical entities detected in stable isotope-resolved metabolomics experiments, especially isotopologues. This was described to the IUPAC Division VIII InChI Subcommittee in 2018 as the “isotopically-resolved spectral feature annotation problem” (Fig. 1). An InChI Isotopologue Working Group was formed to address this challenge, with the following members: Drs. Hunter Moseley (chair), Philippe Rocca-Serra, Reza M. Salek, Masanori Arita, and Emma L. Schymanski. Within six months, the working group developed an initial proposal for extending the InChI standard for representing isotopes within chemical compounds, including isotopomers and isotopologues. The proposal was presented to the IUPAC Division VIII InChI Subcommittee and to the scientific community at Metabolomics 2018, the annual conference of the International Metabolomics Society, in Seattle, Washington USA for feedback and public comment. The original proposal was thus developed into the InChI extension suggested here based on the feedback provided (see supplemental material).

This article presents the InChI Isotopologue and Isotopomer extension, which includes new layer specifications within the InChI standard for representing isotopes within chemical compounds. The extension builds upon the previous isotopic “/i” layer specification, but does not change the specification of this layer. Instead, the interpretation of this layer has been altered to better represent isotopically-resolved chemical information. In addition, a new isotopologue “/a” layer specification has been added to represent isotopologues, i.e., ambiguous isotope localization. The new isotopic “/i” layer interpretation and the new isotopologue “/a” layer specification support the unique identification of features detected and measured in analytical instrumentation corresponding to isotopically-resolved chemical entities. Thus, the new extension enables improved annotation of analytical datasets used for chemical characterization. Such expansion of chemical annotation capabilities will enhance accurate and specific documentation of the isotopic content of chemical features and entities, promoting the FAIR (Findable, Accessible, Interoperable, and Reusable) guiding principles of scientific data stewardship [12] and Open Science [13, 14] with respect to chemical characterization data.

## Chemical terminology

**Fig. 2:**
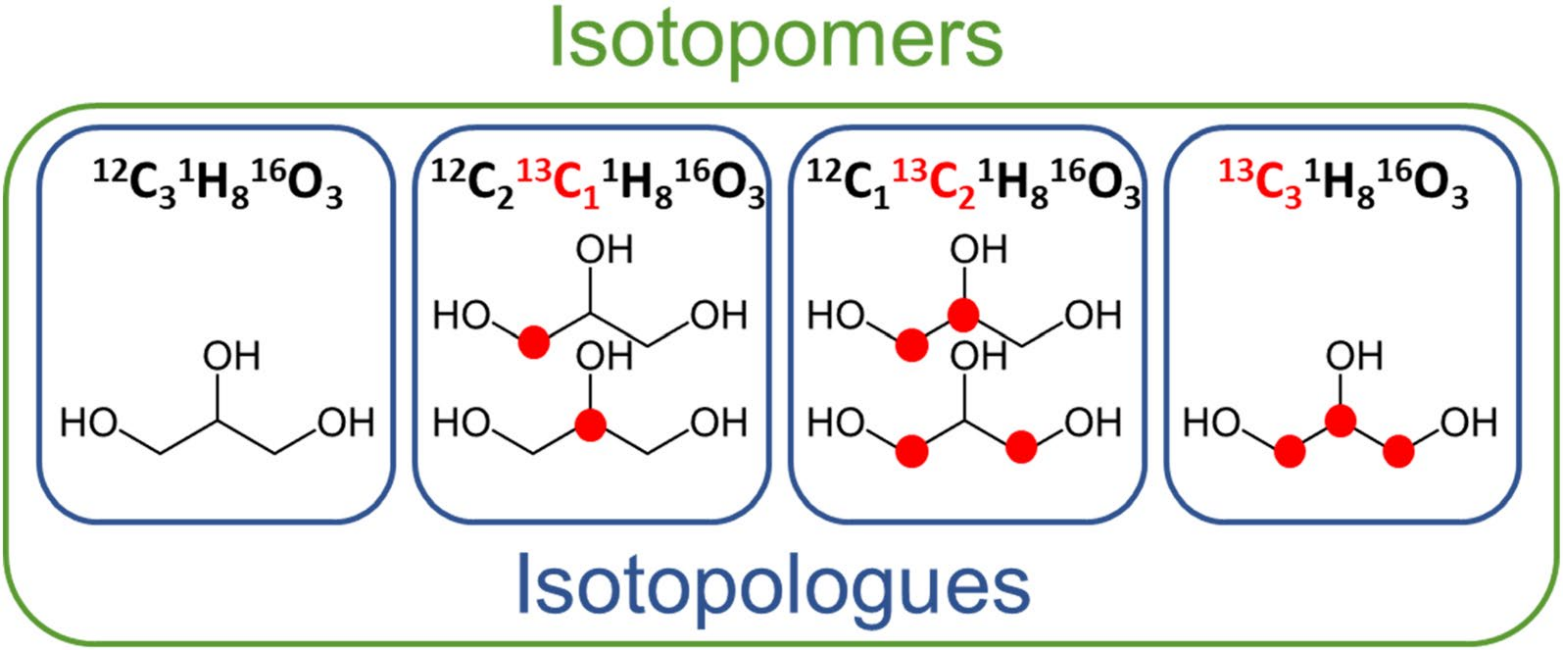
Representation of ^13^C isotopomers and isotopologues in glycerol. The red dot represents the location of ^13^C within the glycerol structure

Specific chemical terminology is needed to understand the new InChI standard specifications and their purpose, which we define below. These definitions draw heavily from definitions provided by the IUPAC Gold Book [15, 16] and Wikipedia. “*Structural isomer*” or “*constitutional isomerism*” is one of a set of isomeric molecules with the same molecular formula but with different bonding patterns and atomic organization (i.e. line formulae) [17, 18]. “*Stereoisomer*” is one of a set of isomeric molecules that have the same molecular formula and sequence of bonded atoms (identical constitution) but differ in the orientation of their atoms in three-dimensional space [19, 20]. “*(Extended) isotopomer*”, also known as an isotopic isomer, is one of a set of isomeric molecules with the same number of each isotope of each element, but differing in their positions within the chemical structure. For example, CH_3_CHDCH_3_ (SMILES: CC([2H])C) and CH_3_CH_2_CH_2_D (SMILES: CCC[2H]) are a pair of “*constitutional isotopomers*” of propane [21, 22]. Isotopomers can also be specific to stereochemistry, where they are known as “*isotopic stereoisomers*” (e.g. (*R*)- and (*S*)-CH_3_CHDOH) [21, 22]. By definition, isotopomers have the same mass, since they have the same isotopic composition. However, the set of all isotopomers, which span all possible isotopic compositions, are often collectively referred to as isotopomers (Fig. 2). While technically inaccurate, it is a pragmatic use of the term to refer to all possible isotopomers spanning all isotopic compositions for a given chemical entity. Therefore, we will refer to “*isotopomer*” as any isotope-position-distinct isomer of a given chemical entity. The term “*mass-equivalent isotopomer*” will be used to refer to the technical definition of isotopomer.

An *“isotopologue*” represents molecules of the same chemical entity and same isotopic composition. As shown in Fig. 2, related isotopologues represent the same chemical entity but differ in their isotopic composition (number of isotopic substitutions) [23, 24]. For example, the isotopologues of water that span the stable isotope compositions of hydrogen and oxygen are: ^1^H_2_^16^O, ^1^H^2^H^16^O, ^2^H_2_^16^O, ^1^H_2_^17^O, ^1^H^2^H^17^O, ^2^H_2_^17^O, ^1^H_2_^18^O, ^1^H^2^H^18^O, and ^2^H_2_^18^O. A single isotopologue represents a set of mass-equivalent isotopomers (e.g. ^12^C_2_^1^H_6_ has one isotopomer; ^12^C_2_^1^H_5_^2^H has two isotopomers). A “*nominal-mass isotopologue*” is a set of isotopologues with the same elemental and nucleon compositions. In many mass spectra, the detected spectral features are not isotopically-resolved but are elementally and nucleon-resolved, thus representing what we will term a nominal-mass isotopologue.

**Fig. 3:**
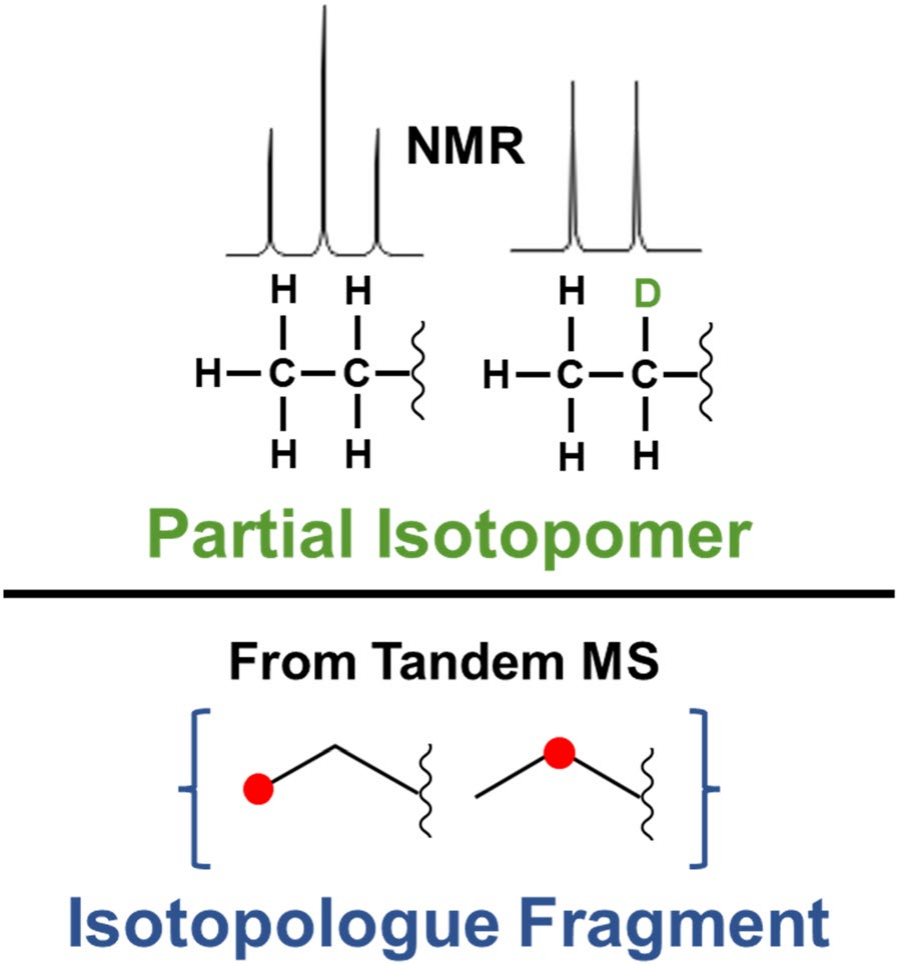
Representation of a partial isotopomer and isotopologue fragment. In NMR, spin–spin coupling (J-coupling or scalar coupling) can produce peaks that indicate the isotopic status of neighboring atoms representing a partial isotopomer. In the top example, the difference between a triplet vs doublet peak pattern indicates a partial isotopomer of H vs ^2^H (D) bonded to the second carbon. In the bottom example, a peak in a tandem MS spectrum represents a single ^13^C in the ethyl fragment of a larger compound

We now introduce two new chemical terms not previously defined to our knowledge before developing this extension to the InChI standard. “*Partial isotopomer*” is the part of an isotopomer where the isotopic content of specific atoms is known. In nuclear magnetic resonance spectroscopy (NMR) and in rare circumstances in tandem mass spectrometry, individual spectral features may directly indicate that certain atoms of a particular molecule have certain isotopes (see Fig. 3). Taking an example from NMR, certain analytical features may indicate that carbon 1 of alpha-d-glucopyranose is ^13^C labeled without knowing the isotopic status of the other carbon atoms. “*Isotopologue fragment*” is a refined set of isotopomers where the ambiguity of isotope location is limited to a subset of the atoms. This is a subset of the mass-equivalent isotopomers representing the full isotopologue. Tandem mass spectrometry may provide spectral features that indicate where (possibly mixed) isotopes are localized within a chemical structure (see Fig. 3). For example, ^13^C_1_ may be limited to the glycerol part of a glycerophospholipid and not the fatty acyl chains.

## InChI isotopic layer

**Fig. 4:**
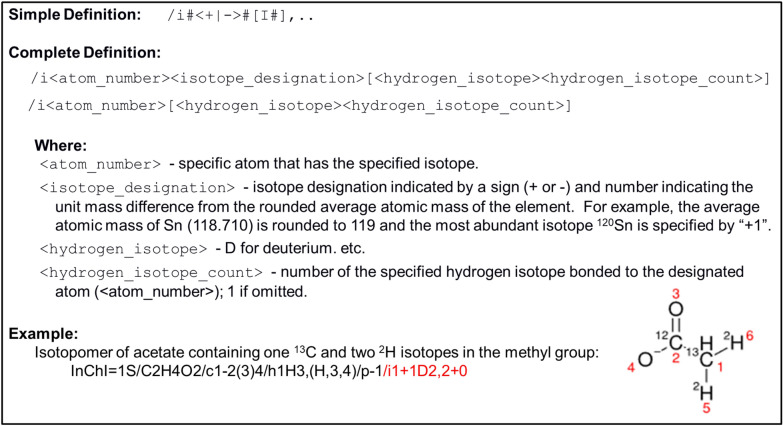
Concise description of the InChI isotopic layer specification. Includes a simple definition, a complete definition with the description of terms, and an example. The example includes the line formula (2D representation) and InChI string of acetate containing one ^13^C and two ^2^H isotopes in the methyl group. The isotopic layer is highlighted in red

The InChI isotopic layer was designed to represent the exact isotopomers of specific compounds. As described in Fig. 4, the isotopic layer starts with a “/i” followed by the atom number based on InChI canonicalization and then the isotope designation and hydrogen isotope designation, if present. This simplifies it to the atom number and hydrogen isotope designation if no isotope designation is needed. Previously, the InChI isotopic layer could only specify exact isotopomers, with limited ability to describe atomic location ambiguity for hydrogen isotopes. Also, the previous interpretation of the isotopic layer for exact isotopomers was minimalistic, assuming that unspecified isotopes were the most abundant stable isotope.

**Fig. 5:**
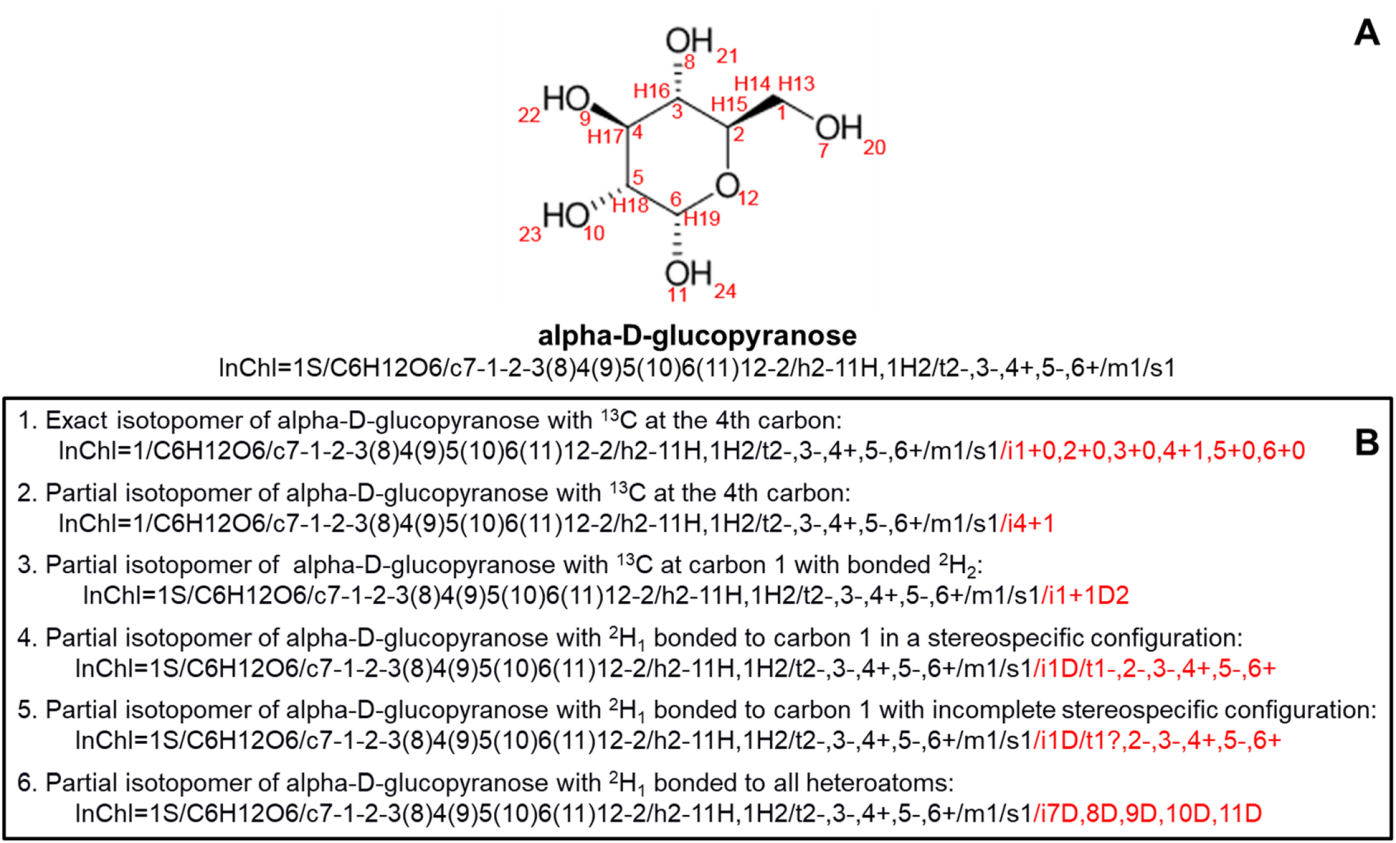
Examples of exact and partial isotopomer InChI strings. **A** Line formula (2D representation) of alpha-d-glucopyranose along with its InChI string. **B** Specific exact and partial isotopomer examples of alpha-d-glucopyranose, with the isotopic layer highlighted in red

The new interpretation of the InChI isotopic layer enables both exact isotopomers and partial isotopomers to be specified. Figure 5 illustrates examples of both exact and partial isotopomer interpretations of the InChI isotopic layer. Exact isotopomers require explicit definition of the isotope for each atom of an element (Fig. 5B(1)). In contrast, a partial isotopomer only requires the isotope specification for a subset of atoms for a given element (Fig. 5B(2, 3)). Furthermore, exact and partial isotopomers can be described in a stereospecific configuration (Fig. 5B(4)). However, as shown in Fig. 5B(5), exact and partial isotopomers with incomplete stereospecific configurations and involving deuterium isotopes can technically represent ambiguity in the location of the deuterium isotope, i.e., an isotopologue or isotopologue fragment. The previous interpretation of the isotopic layer would have interpreted partial isotopomers as exact isotopomers, thus preventing the representation of partial isotopomers. Since the isotopic layer specification remains unchanged, the new interpretation does not require any modifications to the underlying InChI algorithms or software and, thus, is currently implemented. Therefore, partial isotopomers can be specified using the InChI standard, especially for annotating most individual NMR spectroscopy spectral features. Moreover, the final example shown in Fig. 5B(6) demonstrates a partial isotopomer that would arise in H/D exchange experiments, when labile hydrogens are replaced with deuterium isotopes. This example illustrates the varied applications of partial isotopomer annotation in experiments utilizing stable isotopes, made possible by the refined specifications.

## InChI isotopologue layer

To represent isotopologues, a new ambiguous isotope localization “/a” layer specification has been added. The isotopologue layer starts with a “/a” followed by an encoded description within parentheses. However, the specific encoding depends on the type of isotopologue or isotopologue fragment being specified: (a) isotopically-resolved structural isomer, (b) nominal-mass-resolved structural isomer, or (c) with only molecular formula resolution, i.e., unknown or ambiguous structural isomers.

**Fig. 6:**
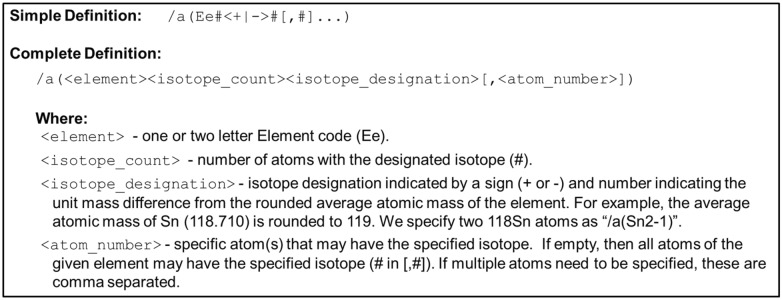
Concise description of the InChI isotopically-resolved isotopologue layer specification of a constitutional isomer. Includes a simple definition and a complete definition with the description of the terms

For isotopically-resolved isotopologues of a constitutional isomer, the encoded description within the parentheses starts with the element code followed by the isotope count or number of atoms with the designated isotope, and then ending with the isotope designation, which is indicated by a sign (+ or −) and number indicating the unit mass difference from the rounded average atomic mass of the element. Isotopically-resolved isotopologue fragments of a constitutional isomer include an additional comma-separated list of atom numbers designating specific atoms where the designated isotope could be. The encoding specification is fully described in Fig. 6.

**Fig. 7:**
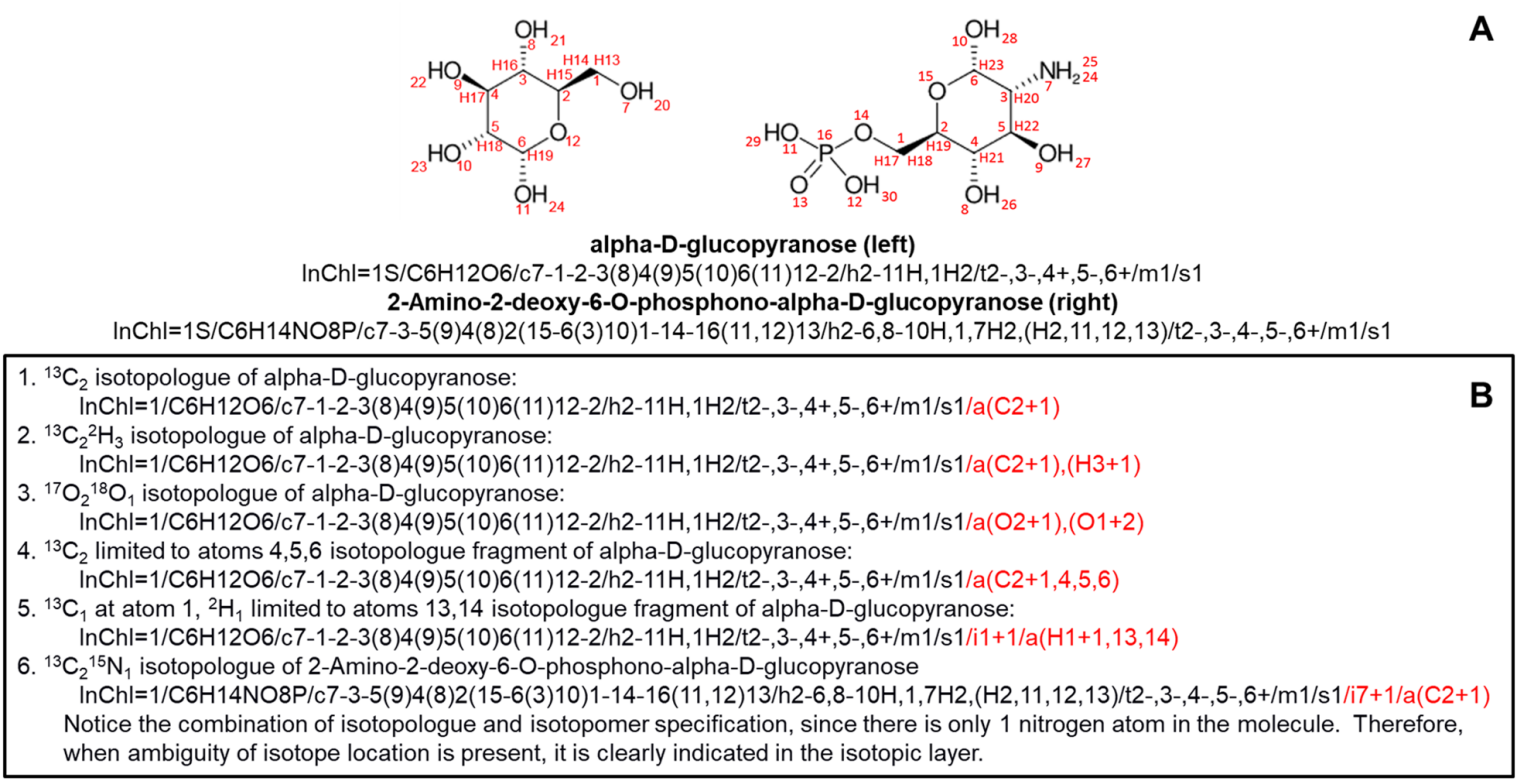
Examples of isotopically-resolved isotopologues and isotopologue fragments. **A** Left is the line formula (2D representation) of alpha-d-glucopyranose along with its InChI string below. Right is the line formula of 2-amino-2-deoxy-6-*O*-phosphono-alpha-d-glucopyranose with its InChI string below. **B** Specific examples of isotopically-resolved isotopologues and isotopologue fragments of these two compounds, with the isotopologue layer highlighted in red

Figure 7 illustrates examples of both isotopologues and isotopologue fragments. Specifically, Fig. 7B(1–3) illustrate ^13^C, ^2^H, and/or ^17^O/^18^O isotopologues of alpha-d-glucopyranose, whereas Fig. 7B(4–6) illustrate ^13^C, ^2^H, and/or ^15^N isotopologue fragments. However, pay attention to Fig. 7B(6) where an isotopic layer must be used to specify the ^15^N_1_ at atom 7 instead of being part of the isotopologue layer, since the molecule has only one nitrogen atom. This is an important boundary condition between using the isotopic vs isotopologue layer for specifying the isotope content. The “/a” isotopologue layer is only used when the location of the isotope is “ambiguous”. Besides the layer designation “/a” vs “/i”, the use of parentheses in the isotopologue layer also makes it visually distinct from the isotopic layer.

**Fig. 8:**
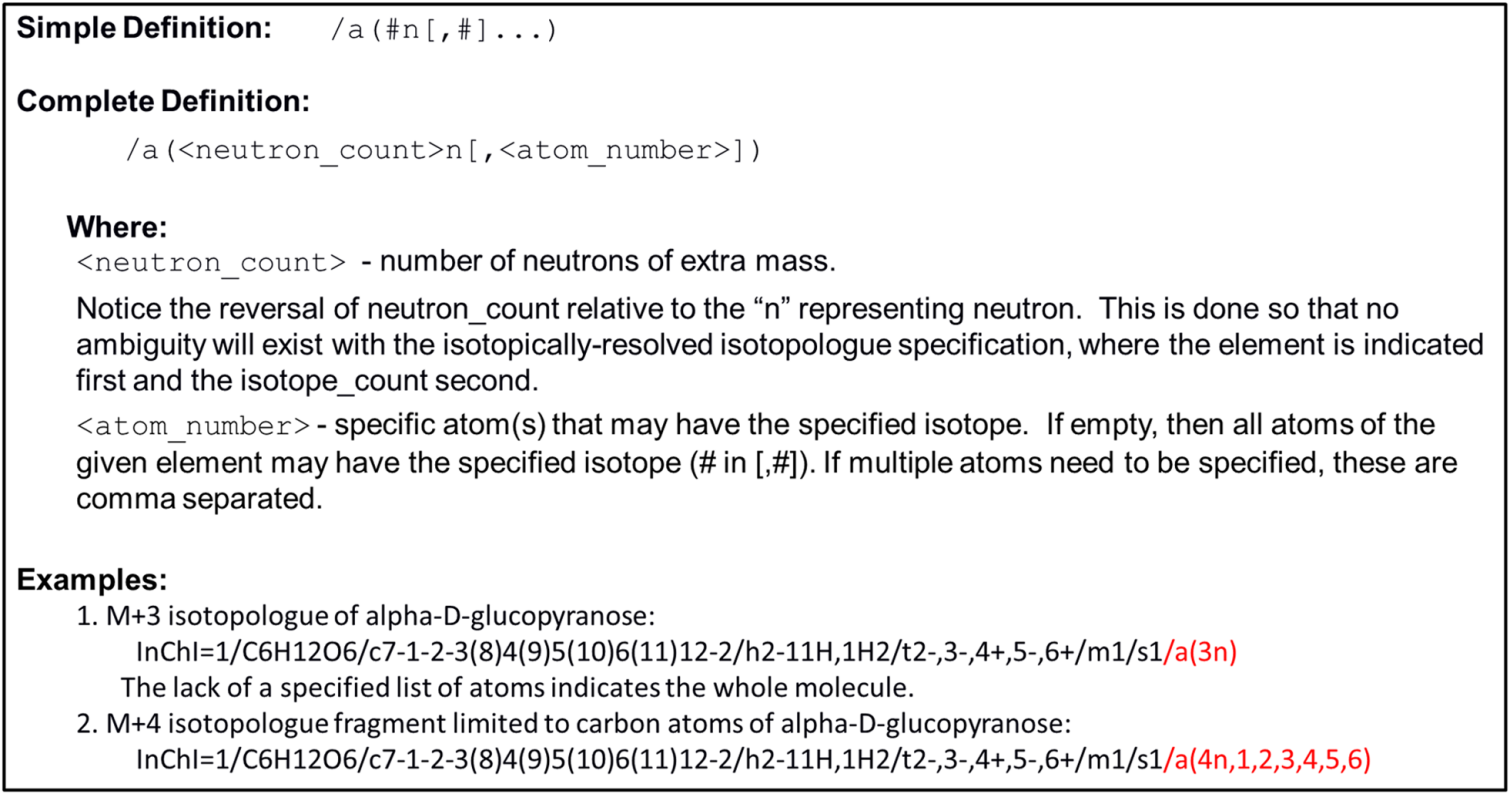
Concise description of the InChI nominal-mass-resolved isotopologue layer specification of a constitutional isomer. Includes a simple definition, a complete definition with the description of the terms, and examples. The example includes the line formula (2D representation) and InChI string of alpha-d-glucopyranose with 3 or 4 extra neutrons. The isotopic layer is highlighted in red

For the nominal-mass-resolved isotopologues of a constitutional isomer, the description within the parentheses begins with the neutron count followed by “n,” as shown in Fig. 8. Nominal-mass-resolved isotopologue fragments of a constitutional isomer include an additional comma-separated list of atom numbers designating specific atoms where a specific neutron could be located. Example 1 in Fig. 8 illustrates a nominal-mass isotopologue with 3 extra neutrons beyond what is expected for the most common stable isotopes present in alpha-d-glucopyranose. Example 2 in Fig. 8 illustrates an isotopologue fragment with 4 extra neutrons across the carbon atoms of alpha-d-glucopyranose.

**Fig. 9:**
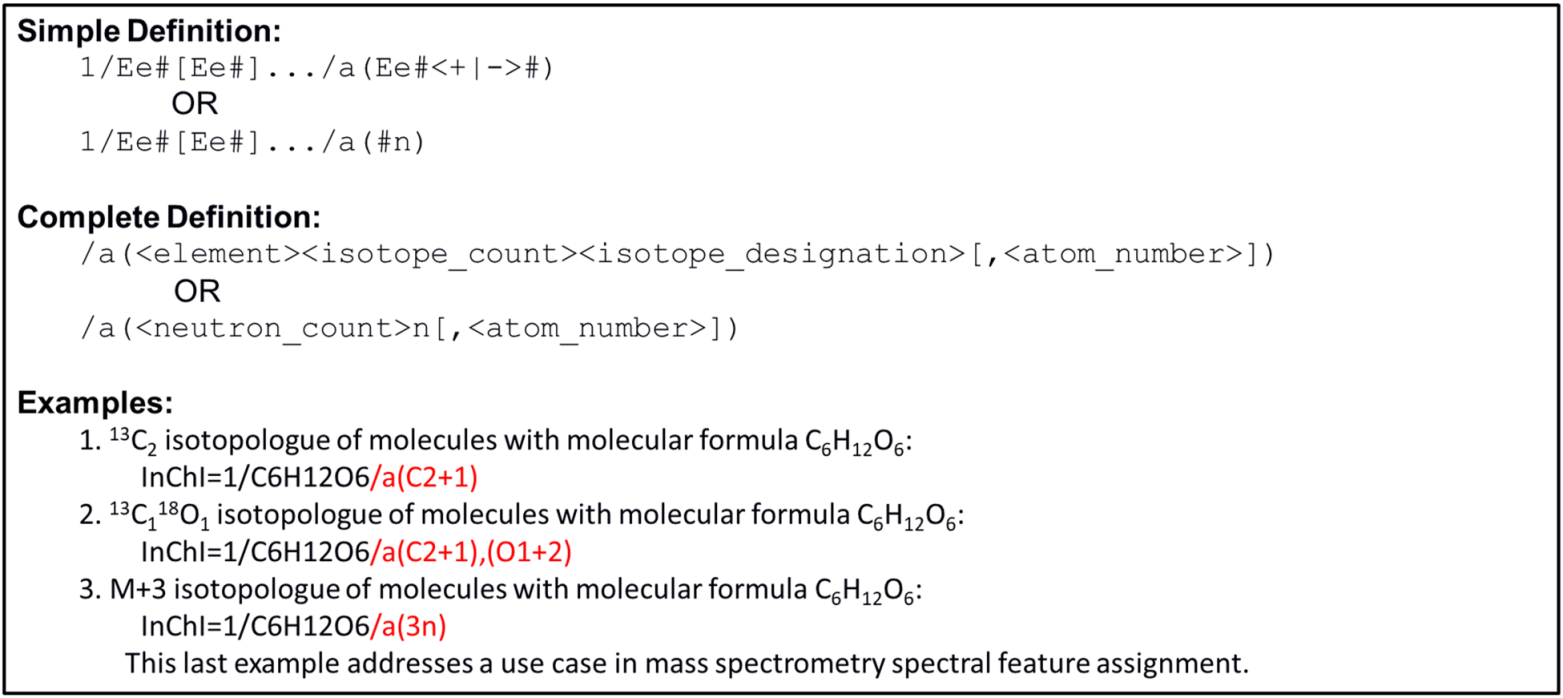
Concise description of the InChI isotopologue layer specification of an unknown constitutional isomer. Includes a simple definition, a complete definition with the description of the terms, and examples. The first two examples involve the molecular formula C_6_H_12_O_6_ with ^13^C and ^18^O isotopes. The last example involves the molecular formula C_6_H_12_O_6_ with 3 extra neutrons. The isotopic layer is highlighted in red

For nominal-mass-resolved isotopologues where the constitutional isomer is unknown (or highly ambiguous), a truncated InChI string with only the molecular formula and the isotopologue layer is used with either an isotopically-resolved or nominal-mass encoded description. Examples 1 and 2 in Fig. 9 illustrate ^13^C and ^18^O containing isotope-resolved isotopologues of the molecular formula C_6_H_12_O_6_, representing 6-carbon saccharides. Example 3 in Fig. 9 illustrates a nominal-mass-resolved isotopologue of the molecular formula C_6_H_12_O_6_ containing 3 extra neutrons above what is expected for the most common stable isotope of the elements present. Due to the lack of chemical (sub)structure with atom identifiers, isotopologue fragments of an unknown constitutional isomer cannot be defined.

## Conclusions

This article details a proposed extension to the InChI standard that allows the representation of isotopically-resolved chemical entities at varying levels of ambiguity in isotope location. This extension includes improved interpretation of the current isotopic “/i” layer for representing exact isotopomers and partial isotopomers. In addition, the extension includes an isotopologue “/a” layer specification for representing chemical entities with ambiguous isotope localization. This new isotopologue “/a” layer enables the representation of isotopologues and isotopologue fragments. Both improvements support the unique identification of features detected and measured in analytical instrumentation, enabling their interpretation as isotopically-resolved chemical entities. Thus, this new extension would enable improved annotation of analytical datasets characterizing chemical entities, and would better support FAIR data sharing and Open Science once extension-aware programs are available.

## Data Availability

All supplemental materials are available at: 10.6084/m9.figshare.7150964. This includes the original proposal, all feedback and responses on the proposal as well as the presentation of the proposal given at Metabolomics 2018, the annual conference of the International Metabolomics Society in Seattle, Washington USA.

## Abbreviations

IUPAC: International Union of Pure and Applied Chemistry
InChI: IUPAC International Chemical Identifier
API: Application programming interface
SMILES: Simplified Molecular Input Line Entry System
FAIR: Findable, Accessible, Interoperable, and Reusable
